# Altered Granger Causal Connectivity of Resting-State Neural Networks in Patients With Leukoaraiosis-Associated Cognitive Impairment—A Cross-Sectional Study

**DOI:** 10.3389/fneur.2020.00457

**Published:** 2020-06-10

**Authors:** Qingli Shi, Hongyan Chen, Qian Jia, Zinan Yuan, Jinfang Wang, Yuexiu Li, Zaizhu Han, Dapeng Mo, Yumei Zhang

**Affiliations:** ^1^Department of Neurology, Beijing Tiantan Hospital, Capital Medical University, Beijing, China; ^2^Department of Neurology, Beijing Pinggu Hospital, Beijing, China; ^3^Department of Neuroradiology, Beijing Tiantan Hospital, Capital Medical University, Beijing, China; ^4^Department of Neurology, General Hospital of The Yang Tze River Shipping, Wuhan Brain Hospital, Wuhan, China; ^5^Department of Rehabilitation Medicine, Beijing Tiantan Hospital, Capital Medical University, China National Clinical Research Center for Neurological Diseases, Center of Stroke, Beijing Institute for Brain Disorders, Beijing Key Laboratory of Translational Medicine for Cerebrovascular Disease, Beijing, China; ^6^State Key Laboratory for Cognitive Neuroscience and Learning, Beijing Normal University, Beijing, China; ^7^Department of Interventional Neuroradiology, Beijing Tiantan Hospital, Capital Medical University, Beijing, China

**Keywords:** resting-state functional MRI, multivariate Granger causality analysis, independent component analysis, leukoaraiosis, cognitive impairment, brain networks

## Abstract

**Background:** The purpose of this study was to provide an imaging reference for the measurement of disease progression, as well as to reveal the pathogenesis of leukoaraiosis (LA).

**Methods:** Eighty-seven subjects were divided into three groups: LA patients with vascular dementia (LA-VaD) (20 subjects: 14 female, 6 male), LA patients with vascular cognitive impairment nondementia (LA-VCIND) (32 subjects: 14 male, 18 female), and normal controls (NC) (35 subjects: 14 male, 21 female). A multivariate Granger causality analysis (mGCA) was applied to the resting-state networks (RSNs) to evaluate the possible effective connectivity within the resting-state networks retrieved by independent component analysis (ICA) from resting-state functional magnetic resonance imaging (rs-fMRI) data.

**Results:** Ten RSNs were identified: the primary visual network, secondary visual network, auditory network, sensorimotor network, anterior default mode network, posterior default mode network, salience network, dorsal attention network, left working memory network, and the right working memory network. Using independent component analysis, significant average *Z* scores were found in the anterior default mode network, salience network, dorsal attention network, and right working memory network between LA-VAD and NC groups. The functional connectivity (FC) strength of the networks was different between the NC, LA-VCIND, and LA-VaD groups. Effective connectivity between RSNs was compensated by either increased or decreased effective connectivity changes in these three groups.

**Conclusions:** The components of resting-state networks kept changing as the disease progressed. Meanwhile, the activation intensity increased at the early stage of LA and decreased as patients' cognitive impairment aggravated. Furthermore, the direction and strength of connections between these networks changed and remodeled differently. These suggest that the human brain compensates for specific functional changes at different stages.

## Introduction

Leukoaraiosis (LA), a term used in diagnostic imaging, is a small-vessel disease and was first introduced by H. Merskey and colleagues in 1987 (1). Many studies have proposed that LA leads to cognitive decline, such as reduction in the cognitive processing speed, executive function, and visual space function (2, 3).

Functional magnetic resonance imaging (fMRI) can be used to measure oxygen saturation and blood flow in the brain. The blood oxygen level-dependent (BOLD) fMRI method is widely used to observe areas of the brain that are active at a given time. Resting-state functional magnetic resonance imaging (rs-fMRI) is frequently employed in neuroimaging investigations.

In recent years, functional connectivity (FC) measures of rs-fMRI have identified a set of spatially coherent patterns in the human brain, namely, resting-state networks (RSNs); it is mainly described as follows (4, 5): default mode network (DMN), visual network (VN), memory network (MeN), motor and sensory networks (MSN), auditory network (AN), salience network (SN), dorsal attention network (DAN), and the executive control network (ECN). These RSNs are involved in multiple cognitive functions, such as episodic memory, vision, movement, hearing, attention, and executive control (6, 7).

Functional interaction of these networks were thought to sustain our daily behavior and emotional activities, and the altered connectivity of the RSNs were associated with cognitive decline in patients with various disorders, such as Alzheimer's disease (8), cerebral small vessel disease (9), and multiple sclerosis (10). Most studies have found changes in connectivity of the RSNs in patients with LA-associated cognitive impairment; some changes may protect against detrimental effects of white matter damage on cognitive functions, and the altered causal connectivity of the RSNs may elucidate the dysfunctional and compensatory processes in LA patients (11–14). The present study aimed to compare the altered patterns in patients with different cognitive impairment loads caused by LA with those of healthy controls. Given the aforementioned studies, we hypothesized that the altered FC patterns would be associated with cognitive impairments in patients with LA, and the FC patterns changes with different cognitive burdens. To better understand the disrupted networks, we combined independent component analysis (ICA) and Granger causality analysis (GCA) to investigate alternations in RSNs. Our study provides new insights into the underlying mechanisms of LA-associated cognitive impairment.

## Materials and Methods

### Ethics Statement

The present study was approved by the Human Ethical Committee of Beijing Tiantan Hospital, Capital Medical University, China, and written informed consent was provided by the participants or their legitimate guardians.

### Study Participants

#### Inclusion and Exclusion Criteria

All enrolled subjects received a head MRI scan between March 2012 and March 2016 in the Beijing Tiantan Hospital. Fifty-two patients with LA were recruited, and 35 normal control (NC) study participants without memory complaints were recruited, with age, sex, and education levels matching those of the patients. The diagnosis of LA was made unanimously by two radiologists who independently evaluated the fluid-attenuated inversion recovery (FLAIR) MRIs visually without the knowledge of the participants' clinical profiles.

Inclusion criteria for the LA-VCIND group were as follows:

1. aged 45–80 years old;
2. clinical dementia rating (CDR) ≥0.5 points;
3. brain MRI conforming to clinical neuroimaging diagnosis of LA;
4. 24 ≤ Mini Mental State Examination (MMSE) < 27 with years of education ≥6, or 20 ≤ MMSE < 24 with years of education years <6, or 17 ≤ MMSE < 21 with years of education = 0; and Montreal Cognitive Assessment (MoCA) < 26;
5. written informed consent.

Inclusion criteria for the LA-VaD group were as follows:

1. CDR ≥ 1; MMSE < 24 with ≥6 years of education, MMSE < 20 with < 6 years of education, or MMSE < 17 with 0 years of education; and MoCA < 22;
2. all other criteria mentioned above for the LA-VCIND group.

Inclusion criteria for the NC group were as follows:

1. MRI showed normal brain structure;
2. CDR = 0; MMSE ≥ 27 with years of education ≥6, or MMSE ≥ 24 with years of education < 6, or MMSE ≥ 21 with years of education = 0; and MoCA ≥ 26;
3. all other criteria mentioned for the LA-VCIND group.

Exclusion criteria of the three groups were as follows:

1. symptoms that comply with the diagnostic criteria for Parkinson's disease, frontotemporal dementia, or Huntington's disease;
2. a history of mental illness;
3. leukoencephalopathy of nonvascular origin;
4. other diseases that lead to cognitive impairment symptoms;
5. taking of drugs that affect cognitive function;
6. disturbance in consciousness, aphasia, and other diseases that affect the neuropsychological examination.

### Clinical Cognitive Assessment

All participants were instructed to complete MMSE (15), MoCA (16), and CDR under the supervision of a physician. The following education-specific reference cutoff values for MMSE scores were used: middle and high, 27; elementary, 24; and illiterate, 21 (17). The cutoff value for cognitive impairment in the MoCA was <26 (16). In addition, one additional point was added to the raw MoCA score when the participant's years of education were fewer than 12 years.

### Data Acquisition

All subjects received MRI scans (SIEMENS 3.0 T, Germany) with their head fixed to avoid head movement. The subjects were asked to stay awake and to keep their eyes closed to avoid any form of thought activity during the rs-fMRI scan. The scan lasted ~8 min and 20 s. Scanning parameters were as follows: repetition time (TR) = 2,000 ms, echo time (TE) = 30 ms, matrix = 64 × 64 mm, field of view = 256 × 256 mm^2^, and flip angle = 90°. Twenty slices parallel to the anterior and posterior commissures (6 mm thick and no gap between slices) were imaged to cover the whole brain. After fMRI scanning, structural images were collected using the high-resolution T1-weighted 3D MRI sequences program (voxel 1 × 1 × 1 mm^3^, no gap, TR = 2,100 ms, TE = 3.25 ms, matrix = 256 × 256 mm, field of view = 230 × 230 mm, flip angle = 10°).

### Data Processing

#### Rest-fMRI Data Preprocessing

Rest-fMRI data were preprocessed using the advanced Processing Assistant for Resting-State fMRI (DPARSF) module of the DPABI pipeline (a toolbox for Data Processing and Analysis of Brain Imaging; http://www.rfmri.org) (18). Briefly, after converting the DICOM files to NIFTI images, the first 10 time points were discarded. Then, slice timing and head motion correction were performed. One participant was excluded from the data analysis due to excessive head movement (exceeded 3.0 mm translation or 3° rotation in any direction). The data were then normalized to the standard Montreal Neurological Institute (MNI) space. After smoothing with a 6-mm full width at half maximum (FWHM) Gaussian kernel, the nuisance signals were removed. The Friston 24-parameter model was utilized to regress out head motion artifacts from the realigned data. The signals from white matter and cerebrospinal fluid were also regressed out to reduce respiratory and cardiac effects. Finally, the residual time series were band-filtered within the frequency range of 0.01–0.10 Hz to remove very low-frequency drift and high-frequency noise.

#### Group ICA

After preprocessing the data, we decided *a priori* to select 10 RSNs: the primary visual network (PVN), secondary visual network (SVN), auditory network (AN), sensorimotor network (SMN), anterior default mode network (aDMN), posterior default mode network (pDMN), salience network (SN), DAN, left working memory network (lWMN), and the right working memory network (rWMN), basically includes all the main RSNs. Group ICA of the fMRI Toolbox (GIFT) was used to decompose the data into functional networks using group spatial independent component analysis (http://icatb.sourceforge.net/) (19). The images were reduced to 40 dimensions using principal component analysis (PCA), and the number of independent components (ICs) was estimated to be 25 using the minimum description length criteria (20). The mean ICs of all subjects, the corresponding mean time courses, and the ICs for each subject were obtained from group ICA separation and back reconstruction (19). The intensity values in each spatial map were converted to *Z* scores to indicate the voxels that contributed most strongly to a particular IC. Voxels with absolute *Z* values >1.5 were considered active voxels of the IC in this study (21). Then, a selection of the components to be retained for further analysis among the 25 estimated ICs was performed using anatomic information. Our selected 10 RSNs corresponded to the cerebral components with the largest spatial correlations with the network templates (22, 23), which contained the main components of the RSNs. We subsequently calculated the average *Z* score of active voxels for each selected RSN.

### Multivariate Granger Causal Analysis

GCA is a method for investigating whether one time series can correctly forecast another (24). This method is based on multiple regression analysis. At an individual level, many studies performed F statistics on the residuals (25). A recent study (26) used signed path coefficients to perform *t* tests at group level statistics. The negative path coefficients were explained as an inhibitory effect (27).

Assuming two time series *X* and *Y*, the paired model is as following:

$$\begin{array}{l} Y_{t}=\sum_{n\;=\;1}^{p}A_{n}X_{\left(t-p\right)}+\sum_{n\;=\;1}^{p}B_{n}Y_{\left(t-p\right)}+CZ_{t}+E_{t}\\ X_{t}=\sum_{n=1}^{p}A_{n}\prime Y_{\left(t-p\right)}\prime +\sum_{n=1}^{p}B_{n}\prime X_{\left(t-p\right)}\prime +C\prime Z_{t}+E_{t}\prime \end{array}$$

*X*_*t*_ and *Y*_*t*_ represent the two times series at time *t*. *X*_(*t*−*p*)_ and *Y*_(*t*−*p*)_ represent the time series at time *t–p, p* representing the number of lagged time points (order). *A*_*n*_ and $A_{n}\prime$ are signed path coefficients. *B*_*n*_ and $B_{n}\prime$ are autoregression coefficients. *E*_*t*_ and $E_{t}\prime$ are residuals. We followed Chen's (26) extended vector autoregression model, which took the covariable *Z*_*t*_ at time *t* into account, instead of regressing them out before GCA.

Assuming that (*Y*_1_, *Y*_2_, ⋯ , *Y*_*n*_) are time series of selected brain regions (*n* regions), the signed path coefficient multivariate GCA is as given below:

$$\begin{array}{l} Y_{1t}=\overset{p}{\underset{i\;=\;1}{\sum }}A_{11}^{i}Y_{1}\left(t-i\right)+\ldots +\overset{p}{\underset{i\;=\;1}{\sum }}A_{1n}^{i}Y_{n}\left(t-i\right)+C_{1}Z_{t}+\varepsilon_{t}\\ \vdots \\ Y_{nt}=\overset{p}{\underset{i\;=\;1}{\sum }}A_{n1}^{i}Y_{1}\left(t-i\right)+\ldots +\overset{p}{\underset{i\;=\;1}{\sum }}A_{nn}^{i}Y_{n}\left(t-i\right)+C_{n}Z_{t}+\varepsilon_{t} \end{array}$$

The coefficients matrix (i.e., effective connectivity matrix) at order *i* (*i* = 1, 2, …, *p*) is as follows:

$$\begin{array}{l} \left(\begin{array}{llll} A_{11}^{i}, & A_{12}^{i}, & \cdots , & A_{1n}^{i}\\ A_{21}^{i}, & A_{22}^{i}, & \cdots , & A_{2n}^{i}\\ \vdots \\ A_{n1}^{i}, & A_{n2}^{i}, & \cdots , & A_{nn}^{i} \end{array}\right) \end{array}$$

Considering the complicated significance of high order, we set the lagged time points at 1 (order = 1).

### Statistical Analyses

The chi-square test was used to evaluate the differences in sex distribution among the three groups. Kendall's *W* test was used to evaluate the difference in education years among the three groups. A one-way analysis of variance (ANOVA) was used to compare the differences in age, incidences of hypertension, diabetes mellitus, dyslipidemia, and histories of smoking and drinking. The cognitive test results, i.e., MoCA scores, were compared using analysis of covariance (ANCOVA) with age and sex as covariances. Subsequently, *post hoc* analyses were performed to compare the differences among each of the three groups. *P* < 0.05 was considered statistically significant.

The average *Z* value within the 10 RSNs was extracted, and the ANCOVA method was used to conduct statistical analyses on the three groups of subjects. The influence of sex and age was controlled. *Post hoc* analyses were performed to compare the differences among the three groups. *P* < 0.05 was considered statistically significant.

After the mGCA coefficients matrix at order = 1 was calculated, the effective connectivity patterns of each group were defined as significant mGCA connectivity within the 10 RSNs after a one-sample *t* test [*P* < 0.05, corrected for false discovery rate (FDR)]. Then, the ANCOVA method was used to conduct statistics on the three groups with age and sex as covariances. The FDR correction was performed for multiple comparisons. Subsequently, FDR-corrected *post hoc* analyses within the effective connectivity with significant ANCOVA results were performed to compare the differences among the three groups.

Based on the effective connectivity patterns of each of the three groups, the “in degree,” “out degree,” and “in + out degree” for every RSNs were calculated to extract information on the temporal relations among the RSNs obtained from mGCA.

- “In degree:” number of Granger causal efferent connections to a node (one of the RSNs) from any other node. This causal flow profile identifies nodes that are the central targets of the network.
- “Out degree:” number of Granger causal afferent connections from a node (one of the RSNs) to any other node. This causal flow profile identifies nodes that are the central sources of the network.
- “In + out degree:” sum of “in degree” and “out degree.”

## Results

### Demographic and Clinical Data of Subjects

No statistical differences (*P* > 0.05) were observed for age, sex, education level, hypertension, diabetes mellitus, or disorders of lipid metabolism among the three groups (Table 1). MMSE and MoCA scores were significantly different among the three groups (*P* < 0.05, Table 1). MMSE and MoCA scores in both the NC and LA groups were higher than those of the LA-VaD group (*P* < 0.05).

**Table 1:** Demographic and clinical data in the different groups.

	**NC** **(*n* = 35)**	**LA-VCIND** **(*n* = 32)**	**LA-VaD** **(*n* = 20)**	***P***
Age (mean ± SD) (years)	62.83 ± 6.98	64.34 ± 9.68	65.70 ± 8.01	0.455
Sex (male/female, %)	14/21, 67%	14/18, 78%	6/14, 43%	0.748
Education (years)	10.34 ± 3.50	9.00 ± 3.78	8.10 ± 3.60	0.077
CDR	0	0.5	1	
MMSE	29.40 ± 1.06	27.88 ± 1.83	23.00 ± 3.84	*P*_1_ = 0.000 *P*_2_ = 0.000 *P*_3_ = 0.000
MoCA (mean ± SD)	26.00 ± 2.51	25.77 ± 2.02	23.18 ± 2.83	*P*_1_ = 0.000 *P*_2_ = 0.000 *P*_3_ = 0.000
Hypertension	71% (22/35)	69% (22/32)	59% (13/20)	0.048
Diabetes mellitus	85% (30/35)	87% (28/32)	80% (16/20)	0.297
Disorder of lipid metabolism	80% (28/35)	69% (22/32)	70% (14/20)	0.461

P1: *intergroup test between NC and LA-VCIND groups*.

P2: *intergroup test between LA-VCIND and LA-VaD groups*.

P3, *intergroup test between NC and LA-VaD groups*.

### Components of the 10 Brain Networks

**Figure 1:**
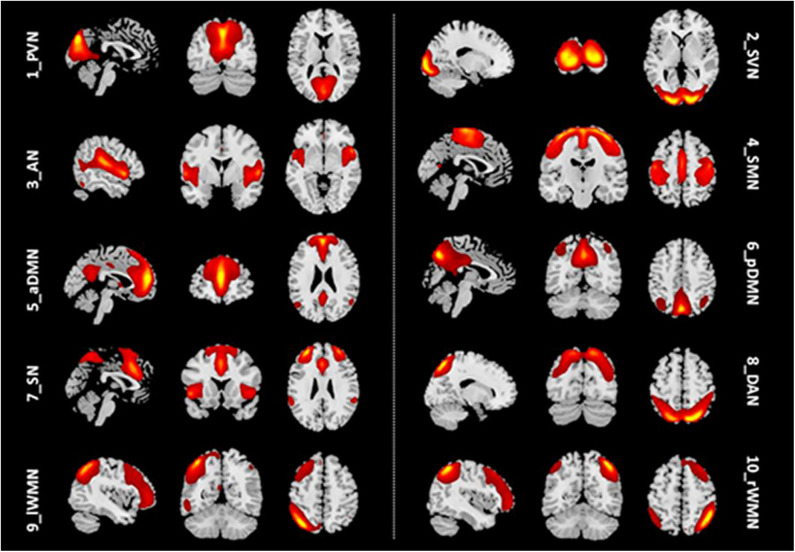
Representation of the 10 RSNs in resting-state functional MRI (fMRI) data of all subjects. The independent components are illustrated as PVN, SVN, AN, SMN, aDMN, pDMN, SN, DAN, lWMN, and rWMN. PVN, primary visual network; SVN, secondary visual network; AN, auditory network; SMN, sensorimotor network; aDMN, anterior default mode network; pDMN, posterior default mode network; SN, salience network; DAN, dorsal attention network; lWMN, left working memory network; rWMN, right working memory network.

We identified 10 ICs for mGCA. The spatial maps of the 10 RSNs selected for effective connectivity analysis in the three groups are illustrated in Figure 1. Table 2 summarizes the components selected in the three groups. On the basis of our results, and those of a large number of RSN studies, the 10 ICs associated with RSNs can be described as follows (Figure 1, Table 2).

**Table 2:** Definition of regions of interest within 10 resting-state networks (RSNs).

**RSNs**	**Core region**	**Number of voxels**	**Peak *Z* value**	**Peak MNI coordinate**
1_PVN	Cuneus, Precuneus, Calcarine, Lingual	1,757	4.3052	0 −81 36
2_SVN	Occipital_Sup, Occipital_Mid, Occipital_Inf, Lingual, Cuneus	1,506	3.1053	−12 −99 −6
3_AN	Temporal_Sup_R, Rolandic_Oper_R, SupraMarginal_R	713	3.1063	63 −21 15
	Temporal_Sup_L, Rolandic_Oper_L	510	3.0213	−63 −24 15
4_SMN	Precentral Gyrus, Postcentral Gyrus, Supp_Motor_Area, Frontal_Sup	1,408	2.6442	−33 −21 69
5_aDMN	Frontal_Sup_Medial, Cingulum_Ant	1,596	4.0204	0 54 15
	Precuneus, Cingulum_Post	123	2.1558	0 −51 21
6_pDMN	Precuneus, Cingulum_Post, Cuneus, Cingulum_Mid	1,977	6.2527	0 −72 39
	Angular_L, Parietal_Inf_L	173	1.7241	−36 −63 54
	Angular_R, Parietal_Inf_R	70	1.3887	39 −63 51
7_SN	Frontal_Mid_L, Frontal_Sup_L, Frontal_Inf_Orb_L, Insula_L	408	3.07	−30 57 18
	Frontal_Mid_R, Frontal_Sup_R, Frontal_Inf_Orb_R, Insula_R	381	2.454	33 54 27
	Cingulum_Ant, Cingulum_Mid, Supp_Motor_Area	299	2.5278	0 24 33
	Precuneus	122	2.0356	−3 −60 60
8_DAN	Parietal_Sup, Parietal_Inf, Precuneus, Occipital_Sup	1,881	4.0973	15 −72 57
9_lWMN	Frontal_Mid_L, Frontal_Inf_L, Frontal_Sup_L	761	2.5129	−42 48 0
	Parietal_Inf_L, Angular_L, Parietal_Sup_L, Precuneus_L	880	5.039	−33 −66 54
10_rWMN	Temporal_Mid_R	104	2.0751	60 −45 −12
	Frontal_Mid_R, Frontal_Sup_R	914	3.3787	33 60 −3
	Parietal_Inf_R, Angular_R, Parietal_Sup_R, SupraMarginal_R	817	5.447	45 −57 54

*RSNs, resting-state networks; MNI, Montreal Neurological Institute; PVN, primary visual network; SVN, secondary visual network; AN auditory network; SMN, sensorimotor network; aDMN, anterior default mode network; pDMN, posterior default mode network; SN, salience network; DAN, dorsal attention network; lWMN, left working memory network; rWMN, right working memory network*.

**Figure 2:**
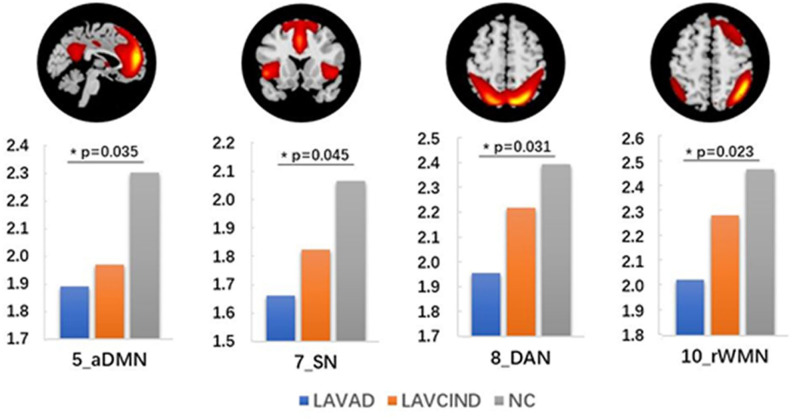
Significant group differences in functional connectivity (FC) (ANCOVA and *post hoc* results, *P* < 0.05).

We compared the average *Z* scores of active voxels for each selected RSN of subjects in the three groups. The average *Z* scores of active voxels for each selected RSN of the three groups are illustrated in Figure 2, Table 3. As shown in Figure 2, the average *Z* scores of active voxels of the aDMN and rWMN were significantly lower in patients with LA-VAD compared with those in NC (*P* < 0.05). Moreover, the average *Z* scores of active voxels of the SN and the DAN in patients with LA-VAD were significantly higher than those in NC (*P* < 0.05).

**Table 3:** Significant group differences in functional connectivity (FC) (ANCOVA and *post hoc* results, * *P* < 0.05).

	***Z*** **value of RSNs (mean** **±** **STD)**			**ANCOVA**		***post hoc*** **(Bonferroni**, ***P*** **value)**		
	**LAVAD (*n* = 20)**	**LAMCI (*n* = 32)**	**NC (*n* = 35)**	***F***	***P***	**LAVAD-NC**	**LAMCI-NC**	**LAVAD-LAMCI**
PVN	2.019 ± 0.970	2.174 ± 0.935	2.372 ± 0.977	0.912	0.406			
SVN	1.898 ± 1.213	2.153 ± 1.037	2.109 ± 0.833	0.424	0.656			
AN	1.708 ± 0.497	1.932 ± 0.456	2.033 ± 0.494	2.917	0.06			
SMN	1.828 ± 0.849	1.675 ± 0.892	1.856 ± 0.731	0.447	0.641			
aDMN	1.891 ± 0.482	1.970 ± 0.710	2.303 ± 0.491	4.221	0.018*	0.035*	0.055	0.881
pDMN	1.817 ± 0.628	2.013 ± 0.679	2.175 ± 0.596	2.047	0.136			
SN	1.661 ± 0.588	1.824 ± 0.707	2.066 ± 0.475	3.202	0.046*	0.045*	0.226	0.603
DAN	1.955 ± 0.717	2.220 ± 0.614	2.396 ± 0.538	3.316	0.041*	0.031*	0.472	0.286
lWMN	2.002 ± 0.686	2.194 ± 0.599	2.358 ± 0.463	2.516	0.087			
rWMN	2.028 ± 0.715	2.287 ± 0.674	2.475 ± 0.415	3.632	0.031*	0.023*	0.401	0.282

### Effective Connectivity Patterns of Subjects Within the Three Groups

**Figure 3:**
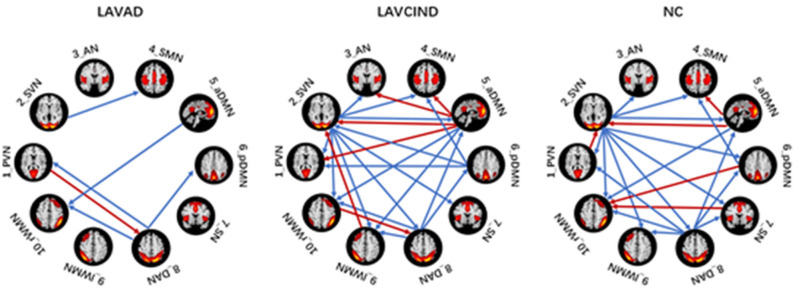
Group differences in functional connectivity (single-sample *t* test, *P* < 0.01).

We used the single-sample *t* test method to show an effective connection with *P* < 0.01. The results are shown in Figure 3, Tables 4–6. The direction of the arrow indicates the direction of the effective connection. A red arrow indicates a positive causal effect, while a blue arrow indicates a negative causal effect. The FC strength of brain networks was different between the NC, LA-VCIND, and LA-VaD groups.

**Table 4:** Functional connectivity of leukoaraiosis patients with vascular dementia (LA-VaD) group (single-sample *t* test, ^*^P < 0.01,^**^P < 0.001).

**Col → Row**	**PVN**	**SVN**	**AN**	**SMN**	**aDMN**	**pDMN**	**SN**	**DAN**	**lWMN**	**rWMN**
PVN	–	−1.992	−0.916	2.504	1.676	−0.834	−0.654	−3.004*	1.250	0.968
SVN	0.257	–	−0.279	2.332	−0.248	0.343	1.098	−1.986	−0.495	1.825
AN	0.074	−1.702	–	0.724	2.826	−2.485	−1.333	2.463	1.139	−0.469
SMN	1.099	−2.875*	1.600	–	1.510	−2.374	−1.707	1.482	0.242	1.520
aDMN	−0.778	−0.665	1.266	0.154	–	1.551	0.652	−1.565	−0.886	2.665
pDMN	1.239	−1.34	0.240	2.211	−0.283	–	0.690	**−5.036** **	−0.309	1.773
SN	1.085	−2.438	0.313	1.234	−1.252	0.477	–	−1.743	1.398	0.629
DAN	3.937**	−2.757	−0.701	1.196	0.042	−0.774	0.917	–	0.287	2.017
lWMN	0.512	−0.346	−0.235	1.372	−1.328	2.116	−0.064	−2.717	–	0.875
rWMN	0.287	−0.948	0.545	0.761	–3.014*	2.639	1.572	–2.938*	1.040	–

*The values in the table represent the T value. The bold values represent those significant ones*.

**Table 5:** Functional connectivity of leukoaraiosis patients with vascular cognitive impairment nondementia (LA-VCIND) group (single-sample *t* test, ^*^P < 0.01, ^**^P < 0.001).

**Col → Row**	**PVN**	**SVN**	**AN**	**SMN**	**aDMN**	**pDMN**	**SN**	**DAN**	**lWMN**	**rWMN**
PVN	–	−3.918**	0.387	1.525	4.309**	–3.106*	−1.689	0.463	0.725	1.660
SVN	2.674	–	0.566	1.109	3.874*	−3.015*	−0.610	−0.440	3.163*	2.013
AN	−0.216	−3.557*	–	2.414	3.104*	−2.171	−1.830	1.848	1.431	−1.180
SMN	1.582	−3.323*	1.677	–	3.716*	−3.845*	−1.409	0.627	1.563	0.884
aDMN	1.795	−3.885*	−0.042	0.755	–	1.513	−0.607	−2.919*	1.008	2.054
pDMN	2.206	−2.621	−0.534	1.198	−0.950	–	−1.044	−2.840*	−0.819	1.992
SN	2.023	−4.093**	1.354	1.545	−0.871	1.092	–	−1.788	0.847	0.941
DAN	2.441	−4.171**	−0.988	0.540	−0.153	−1.367	0.142	–	0.538	3.076*
lWMN	2.690	−3.726*	−0.437	1.412	−2.835*	1.182	0.483	−3.260*	–	1.840
rWMN	2.274	−3.764*	0.510	0.967	−4.043*	2.466	0.451	−4.214*	1.373	–

*The values in the table represent the T value. The bold values represent those significant ones*.

**Table 6:** Functional connectivity of normal control (NC) group (single-sample *t* test, ^*^P < 0.01,^**^P < 0.001).

**Col → Row**	**PVN**	**SVN**	**AN**	**SMN**	**aDMN**	**pDMN**	**SN**	**DAN**	**lWMN**	**rWMN**
PVN	–	−5.849**	0.418	2.436	2.338	−1.974	0.263	−2.967*	1.707	0.884
SVN	3.116*	–	2.003	1.598	3.838*	−1.034	0.202	−2.030	1.443	1.962
AN	1.248	−3.752**	–	2.517	2.237	−1.969	−0.965	−0.517	1.896	−0.138
SMN	1.295	−3.159*	1.676	–	3.053*	−2.897*	−1.789	−0.669	1.902	1.082
aDMN	1.831	−5.127**	1.770	0.826	–	2.050	−0.109	−3.858**	1.697	2.506
pDMN	2.551	−5.688**	2.103	1.949	−1.043	–	0.478	5.481**	1.610	1.346
SN	1.327	−4.287**	1.598	1.934	−1.197	1.998	–	−3.591**	1.686	−0.22
DAN	2.539	−4.695**	1.133	2.188	−1.200	1.005	0.841	–	2.606	1.382
lWMN	2.64	−4.58**	0.950	1.427	−2.226	2.010	1.246	−5.848**	–	0.495
rWMN	1.591	−4.974**	2.081	1.937	−3.713**	3.401*	2.804*	−5.546**	1.877	–

*The values in the table represent the T value. The bold values represent those significant ones*.

**Figure 4:**
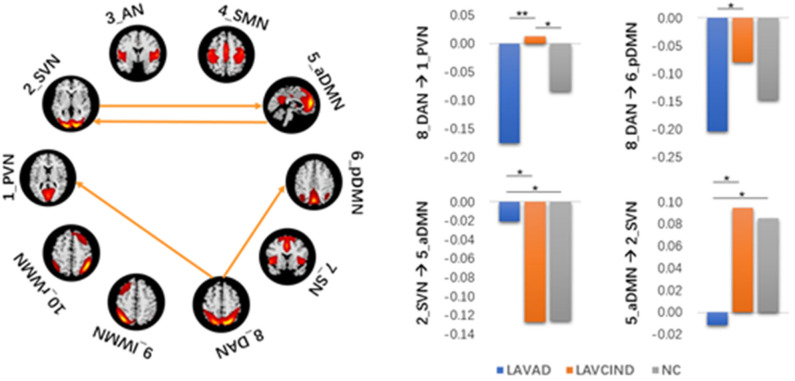
Statistical group differences in intra- and internetwork connectivity at the network level (ANCOVA results, sex and age controlled, **P < 0.01, *P < 0.05).

The ANCOVA method was then used to conduct statistics on the three groups. As seen in Figure 4 and Table 7, the altered connectivity was mainly between the DAN and PVN, SVN and aDMN, aDMN and SVN, and DAN and pDMN. Compared with the LA-VCIND group, the DAN had weaker causal interactions with the pDMN in the LA-VaD group. Compared with the NC and LA-VaD groups, the DAN had strong causal interactions with the PVN in the LA-VCIND group. The casual interactions between SVN and aDMN was significantly different in the LA-VaD group than that in NC and LA-VCIND groups.

**Table 7:** Statistical group differences in intra- and internetwork connectivity at the network level (ANCOVA results, sex and age controlled, ^**^P < 0.01, ^*^P < 0.05).

	**Coefficients (mean** **±** **STD)**			**ANCOVA**		***post hoc*** **(Bonferroni**, ***P*** **value)**		
	**LAVAD (*n* = 20)**	**LAMCI (*n* = 32)**	**NC (*n* = 35)**	***F***	***P***	**LAVAD-NC**	**LAMCI-NC**	**LAVAD-LAMCI**
DAN → PVN	−0.175 ± 0.261	0.012 ± 0.15	−0.083 ± 0.166	6.360	0.003**	0.118	0.016*	0.002**
DAN → pDMN	−0.203 ± 0.181	−0.079 ± 0.158	−0.147 ± 0.158	3.709	0.029*	0.228	0.086	0.012*
SVN → aDMN	−0.021 ± 0.14	−0.127 ± 0.186	−0.126 ± 0.145	3.359	0.040*	0.012*	0.972	0.032*
aDMN → SVN	−0.012 ± 0.216	0.095 ± 0.138	0.085 ± 0.131	3.273	0.043*	0.042*	0.768	0.034*

**Figure 5:**
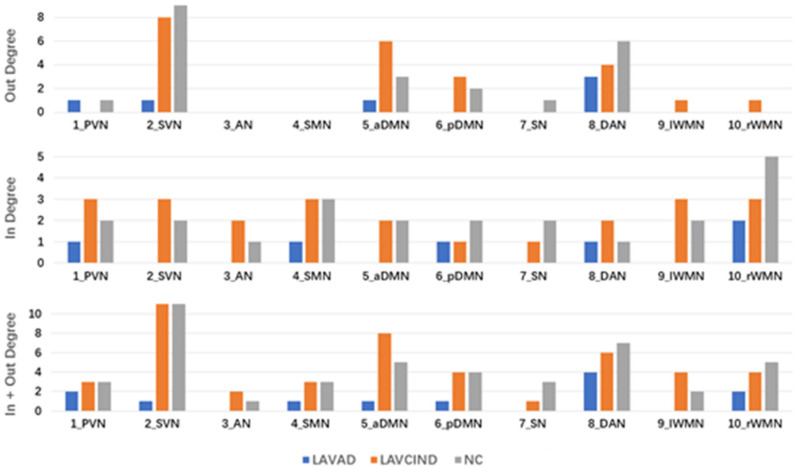
The “in degree,” “out degree,” and “in + out degree” of each resting-state network (RSN) in the two multivariate Granger causality analysis networks. A node with high “in degree” can be considered to be the central target of the network, whereas a node with high “out degree” can be considered to be the central source of the network. A node with “in + out degree,” with at least one standard deviation greater than the average “in + out degree” for all RSNs was identified as a hub in the network.

To better evaluate the causal interactions among RSNs, we show the “in degree,” “out degree,” and “in + out degree” for every RSN in the two networks in Figure 5. In the NC group, the mean “in + out degree” for every RSN was 4.4 and the standard deviation (SD) was 2.88. The SVN and DAN served as hubs according to the standard, the rWMN as central targets, and the DAN as central sources. In the LA-VCIND group, the mean “in + out degree” for every RSN was 4.6 and SD was 2.99. The SVN was identified as the central source, and the PVN/SVN/SMN/WMN served as central targets in this group. Meanwhile, in the LA-VaD group, the mean “in + out degree” for every RSN was 4.4 and SD was 2.88. The SVN served as central sources, whereas the rWMN served as central targets in the network in LA patients with dementia.

## Discussion

The present study used ICA and mGCA to assess functional and effective connections between RSNs in patients with LA, with different levels of cognitive impairment, and healthy controls. The major findings were as follows: (1) FC strength of brain networks was different between the NC, LA-VCIND, and LA-VaD groups; (2) effective connectivity between RSNs was compensated by either increased or decreased effective connectivity changes in these three groups.

In our study, we found that FC strength of brain networks was different between the NC, LA-VCIND, and LA-VaD groups. Effective connectivity between RSNs was compensated by either increased or decreased effective connectivity changes in these three groups.

ICA successfully identified the resting-state components in LA patients and normal control subjects. We were then able to examine the causality interactions among these RSNs and identify their effective connectivity using mGCA. We isolated 10 RSNs, namely, the PVN, SVN, AN, SMN, aDMN, pDMN, SN, DAN, left WMN, and R-WMN, which were not completely consistent with those defined by previous neuroimaging studies (4–6, 28, 29). This variation could be due to different methods with other studies, and the naming of the RSNs may be different with other studies (e.g., the working memory network we defined here, was named as the executive control network, or frontal–parietal network/central executive network in other studies, but the main nodes are the frontal lobe and the posterior parietal lobe). In addition, the network we choose basically includes all the main RSNs. Although the scope of the network defined in each study is different, the main nodes are consistent.

The DMN is associated with episodic memory (30), the DAN with attention, and the SN with cognitive information processing. The presence of white matter hyperintensities (WMH) was significantly associated with concurrent cognitive deficits in all examined domains: general intelligence, memory, processing speed, attention and executive functions, and perception/construction. The progression of WMHs was associated with even worse cognitive functioning, most pronounced in attention and executive functioning (31). Consistent with these clinical features, our data showed decreased *Z* scores in the aDMN, SN, DAN, and rWMN in the LA-VaD group compared with the NC group. This suggests that spontaneous activity of the cognitive-associated network decreased with the decline of cognitive function in LA patients.

There was no obvious positive association between the severity of WML and cognitive decline in some individuals during clinical observations, which may be associated with the individual's cognitive reserve (32) the reorganization between the RSNs (11). In our study, in terms of FC results, we found that the effective connectivity between RSNs decreased significantly with increased cognitive impairment. Previous studies revealed reduced FC in the SMN-AN, SMN-visual network, frontal–parietal control network (FPCN)-AN, and DAN-visual network pairs. The increased functional connectivity in the DMN-AN, DMN-FPCN, and DAN-FPCN pairs may reflect functional network reorganization after damage to the white matter (33). Consistent with this, we found that, compared with the LA-VCIND group, the DAN had weaker causal interactions with the pDMN in the LA-VaD group, which may explain the impairments in attention and information retrieval associated with LA.

Some studies have investigated the automatic processes of neuroplasticity triggered by damage of neural tissue (34), which presents as increased or decreased levels of network-to-network interaction (35). These changes are compensatory to maintain function in the face of adverse events associated with aging, such as cerebral atrophy (36) and subcortical white matter damage. These compensatory mechanisms maintain function, but as WMHs accumulate, this compensatory is no longer effective, and in combination with the depleted cognitive reserve, this results in declined cognitive function. As demonstrated in our study, compared with the NC and LA-VaD groups, the DAN had strong causal interactions with the PVN in the LA-VCIND group. This may suggest an enhanced compensatory response of network connection in the early stage in development of cognitive impairment in LA patients, and this compensatory response may be impaired in the progression of VCIND to VaD.

Compared with NC and LA-VCIND groups, the casual interactions between SVN and aDMN was significantly different in the LA-VaD group. The altered connection between the SVN and aDMN appears as a compensatory response to dementia.

In conclusion, the present study revealed widely altered patterns of intranetwork connectivity among the RSNs in patients with different loads of cognitive impairment in LA patients and NC subjects. We demonstrated that FC is significantly associated with cognitive impairment in LA patients. Our findings suggest that the reduced FC were located with the DAN and pDMN, as well as the aDMN and SVN in the LA-VaD group. These alterations may be accompanied by a decline in cognition and attention in patients with LA. The increased FC between the DAN and PVN, and SVN and aDMN may reflect the reorganization of functional networks to compensate for the cognitive impairments associated with LA. These connectivity pattern alterations and their associations with cognitive performance may play a vital role in understanding the association of LA with cognitive and attention decline.

## Data Availability Statement

All datasets generated for this study are included in the article/supplementary material.

## Ethics Statement

The studies involving human participants were reviewed and approved by the Human Ethical Committee of Beijing Tiantan Hospital, Capital Medical University, China. The patients/participants provided their written informed consent to participate in this study.

## Author Contributions

QS and JW carried out the experiments and participated in the sequence alignment. QS drafted the manuscript. HC carried out the brain MRI data acquisition. QS, ZY, YL, and ZH participated in the brain imaging data analysis. QS and YZ participated in the design of the study and performed the statistical analyses. YZ conceived the study, participated in its design and coordination, and helped draft the manuscript. DM conceived the study, participated in its design and coordination, and helped draft and revised the manuscript. QJ participated in the brain imaging data analysis and revised the manuscript. All authors read and approved the final manuscript.

## Conflict of Interest

The authors declare that the research was conducted in the absence of any commercial or financial relationships that could be construed as a potential conflict of interest.
